# An Unusual Presentation of Occult Small-Cell Neuroendocrine Carcinoma as Acalculous Cholecystitis With Widespread Liver Metastasis

**DOI:** 10.7759/cureus.45706

**Published:** 2023-09-21

**Authors:** Kacper Kubiszewski, Parker Hunsaker, Yelena Piazza, Dhruv Patel, Vladimir Neychev

**Affiliations:** 1 Medical School, University of Central Florida College of Medicine, Orlando, USA; 2 Pathology, University of Central Florida College of Medicine, Orlando, USA; 3 Pathology, University of Central Florida Lake Nona Hospital, Orlando, USA; 4 Radiology, University of Central Florida College of Medicine, Orlando, USA; 5 Radiology, University of Central Florida Lake Nona Hospital, Orlando, USA; 6 Surgery, University of Central Florida College of Medicine, Orlando, USA; 7 Surgery, University of Central Florida Lake Nona Hospital, Orlando, USA

**Keywords:** hyperbilirubinemia, small-cell lung carcinoma, lactic acidosis, liver metastatic disease, transaminitis, acute cholecystitis, small-cell neuroendocrine carcinoma

## Abstract

Small-cell neuroendocrine carcinoma, often classified as small-cell lung carcinoma (SCLC) type, is an aggressive neuroendocrine tumor with early metastatic potential that can lead to unexpected patient presentations. We report the case of a 69-year-old man who presented to the emergency department with worsening right upper abdominal pain, nausea, and vomiting for the past several days. The clinical picture and the workup, including the complete metabolic panel and complete blood count, were highly suggestive of acute cholecystitis with transaminitis and direct hyperbilirubinemia. The ultrasound and magnetic resonance cholangiopancreatography of the abdomen revealed a diffusely hyperdense and hypertrophic liver without evidence of choledocholithiasis. After initial resuscitation, the patient underwent laparoscopic cholecystectomy. Intraoperative findings were consistent with diffuse miliary liver metastatic disease of unknown etiology, rigid liver parenchyma, an extremely frail gallbladder wall, and mild ascites. A biopsy of the liver and cholecystectomy were performed. The final pathology revealed metastatic SCLC to the liver and widespread intravascular tumor emboli, causing diffuse ischemia of the entire gallbladder wall. The patient’s postoperative course was marked by the development of foudroyant liver insufficiency and worsening severe type B lactic acidosis.

## Introduction

Direct hyperbilirubinemia is frequently encountered by clinicians and usually points to cholestasis. The differential diagnosis is broad, encompassing a wide range of extrahepatic and intrahepatic biliary tree disorders [1]. Among the most common disease processes on the differential are choledocholithiasis or advanced cholecystitis [2].

Neuroendocrine carcinomas (NECs) are high-grade, poorly differentiated malignancies characterized by a very aggressive, insidious behavior of early metastasis and poor clinical prognosis, resembling that of small-cell lung carcinoma (SCLC) [3]. It is estimated that 70% of patients with NEC present with widespread metastatic disease at the time of diagnosis [4]. Common metastatic sites include the bone (20-25%), liver (20-30%), and brain (15-20%), suggesting that the symptomatology and presentation of these patients can be diverse [4]. We describe an atypical case of a 69-year-old man who presented with acalculous cholecystitis, which was found to be secondary to widespread hepatic metastasis of previously undiagnosed NEC.

## Case presentation

A 69-year-old man with a past medical history of cerebrovascular accident, congestive heart failure, and hypertension was seen by his primary care physician for postprandial right upper quadrant abdominal pain, nausea, and vomiting for the past seven days. He was found to have transaminitis, hyperbilirubinemia, and positive sonographic Murphy’s sign on an outpatient ultrasound (US) scan of the abdomen. He was sent to the emergency department (ED) for further management. The patient’s home medications consisted of ascorbic acid 1,000 mg once daily, atorvastatin 80 mg once nightly, clopidogrel 75 mg once daily, cyanocobalamin 1,000 µg once daily, lisinopril 10 mg once daily, and an iron-containing multivitamin tablet once daily. There was no reported history of prior abdominal surgeries. He denied current alcohol or recreational drug use but endorsed smoking one pack of cigarettes per day for approximately 30 years, for which he had been screened with yearly chest computerized tomography (CT) scans. His last chest CT and labs, including a comprehensive metabolic panel (CMP) and complete blood cell count (CBC) three months prior, were unremarkable, and he was told to follow up in another year for his next screening.

The physical examination in the ED revealed a jaundiced and afebrile (36.9°C) patient in no acute distress, with a blood pressure of 144/75 mmHg, pulse rate of 84 beats per minute, oxygen saturation of 98% on room air, and respiratory frequency of 18 breaths per minute. His abdomen was soft and non-distended with moderate-to-severe tenderness to palpation in the right upper quadrant with positive Murphy’s sign.

The CMP was notable for direct hyperbilirubinemia (total bilirubin 2.7 mg/dL and direct fraction 2.1 mg/dL). His aspartate transaminase (AST), alanine transaminase (ALT), and alkaline phosphate were elevated (297 U/L, 273 U/L, and 554 U/L, respectively). His serum lipase was 199 U/L. There was a borderline low bicarbonate of 21.0 mmol/L with an anion gap of 19.2 mmol/L. The CBC revealed mildly elevated leukocytes (11.7 x 10^3^/µL) and mild normocytic anemia (red blood cell count 4.54 x 10^6^/µL, hemoglobin 12.9 g/dL, hematocrit 40.0%, and mean corpuscular volume 88.1 fL). His platelet count was normal (170 x 10^3^/µL).

**Figure 1:**
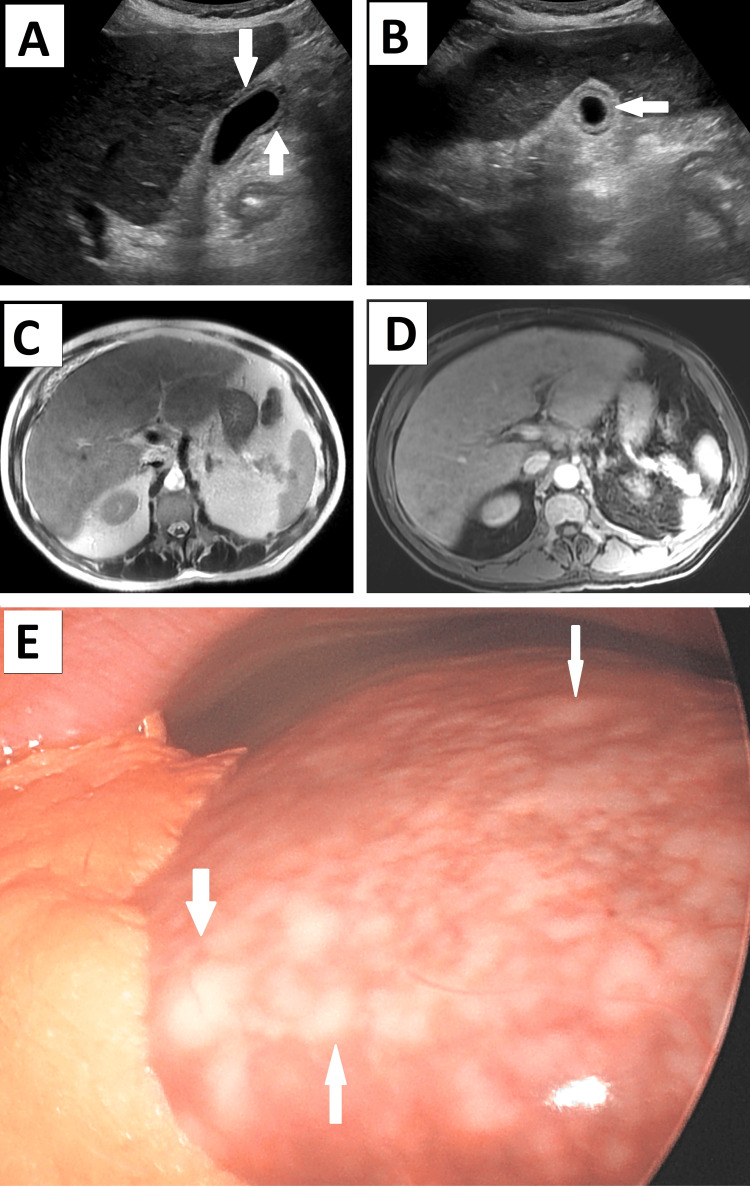
Preoperative imaging and intraoperative findings. (A) Sagittal and (B) transverse view of the ultrasound scan of the abdomen with arrows pointing at the thickened gallbladder wall with intramural edema and pericholecystic fluid; (C) T2 axial MRI image and (D) T1 axial view of the T2 phase of the MRI of the abdomen and pelvis showing diffuse heterogeneous liver hypertrophy without clear evidence for discrete lesions/masses; and (E) intraoperative view of the liver with arrows pointing at diffuse miliary whitish masses of different caliber throughout the surface of the liver.

An US of the gallbladder was performed, revealing mild-to-moderate hepatomegaly with gallbladder wall thickening and no choledocholithiasis (Figures 1A, 1B). Magnetic resonance cholangiopancreatography confirmed the hepatomegaly with diffuse heterogeneous enhancement of the liver without any discrete masses, trace ascites with pericholecystic fluid, and no evidence of choledocholithiasis (Figures 1C, 1D). Given the patient’s presentation and workup, the diagnosis of acute-on-chronic cholecystitis was made, and emergent laparoscopic cholecystectomy with indocyanine green fluorescent intraoperative cholangiography was performed. The initial intraoperative inspection of the abdomen revealed mild ascites. The liver parenchyma was virtually replaced by diffuse small white-pinkish lesions suspicious for micronodular cirrhosis versus widespread metastasis (Figure 1E). Biopsy samples were obtained from segments four and five of the liver. During the cholecystectomy, the liver was found to be heavy and rigid, and the gallbladder wall was grayish, unusually frail, and easy to tear. The case was completed as planned and no complications occurred.

The following day, the patient was found more confused compared to his baseline. His bilirubin levels remained unchanged, but both AST (297 U/L to 545 U/L) and ALT (273 U/L to 298 U/L) worsened. He was also found to have a severely elevated lactic acid level of 7.4 mmol/L. Arterial blood gas (ABG) analysis was performed, revealing a pH of 7.34, partial pressure of carbon dioxide (pCO_2_) of 30.0 mmHg, and partial pressure of oxygen (pO_2_) of 71.4 mmHg with a calculated base excess of -8.5 mmol/L. His lactic acid levels trended up throughout his hospital course and gradually reached a peak of 16.2 mmol/L.

**Figure 2:**
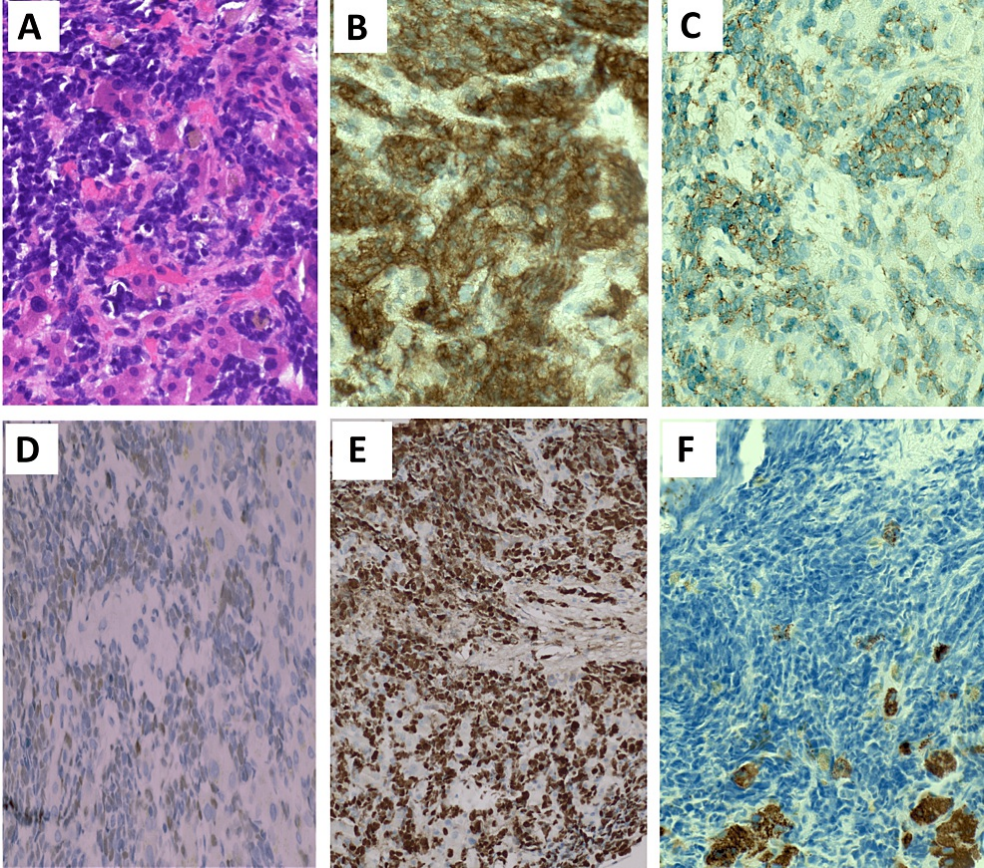
Immunohistochemistry of liver biopsy specimens. (A) Representative high-power image (×400 hematoxylin and eosin) of the tumor; (B) representative high-power (×400) image of the tumor positive for CD56 staining; (C) representative high-power (×400) image of the tumor positive for synaptophysin staining; (D) representative high-power (×400) image of the tumor weakly positive for TTF1 staining; (E) representative high-power (×200) image of the tumor staining for Ki-67 with high mitotic rate of nearly 100%; and (F) representative high-power (×400) image of small, spared areas of uninvolved liver parenchyma positive for Hep Par-1 (brown) surrounded by tumor negative for Hep Par-1 (blue).

**Figure 3:**
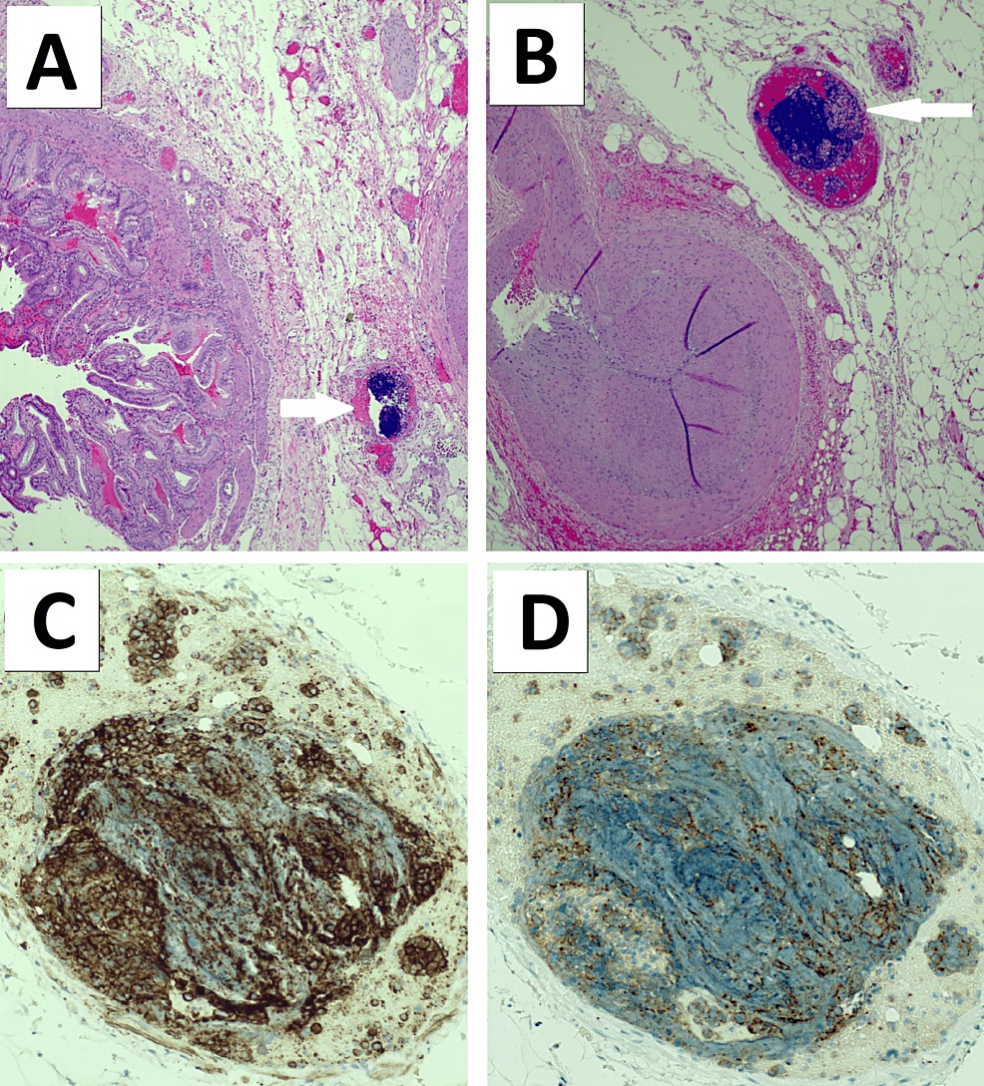
Histopathology and immunohistochemistry of the gallbladder specimen. A) Representative low-power and (B) high-power hematoxylin and eosin image of the gallbladder wall with arrows pointing at diffuse tumor emboli throughout the branches of the cystic artery; (C) representative high-power image of a vascular tumor embolus positive for CD56 staining; and (D) representative high-power image of a vascular tumor embolus positive for synaptophysin staining.

**Figure 4:**
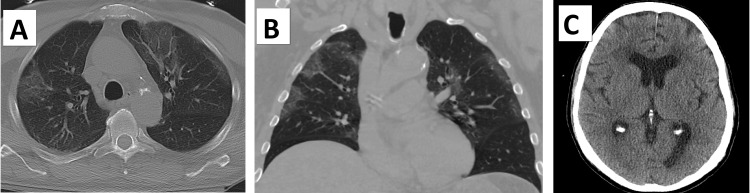
Postoperative imaging. (A) Representative axial and (B) coronal views of a thoracic CT scan demonstrating airspace infiltrates and ground-glass opacities in both upper lobes and the right middle lobe, with no evidence of a discrete tumor; and (C) representative axial CT scan of the brain with no evidence of metastasis to the brain.

The final surgical pathology of the liver biopsies revealed NEC positive for CD56, TTF1, and synaptophysin, with a Ki-67 of nearly 100%, and Hep Par-1 negative consistent with a diagnosis of metastatic small-cell NEC (Figures 2A-2F). The gallbladder pathology revealed widespread intramural, intravascular tumor emboli with diffuse ischemic changes (Figures 3A-3D). A postoperative CT of the chest was performed on postoperative day four, which revealed airspace infiltrates and ground-glass opacities in the upper lobes and right middle lobe, but no evidence of a discrete tumor (Figures 4A, 4B). A CT of the brain showed no evidence of brain metastasis (Figure 4C). The patient’s condition continued to deteriorate despite full-range resuscitative and supportive measures. There was no clinical or paraclinical evidence of an ongoing intra-abdominal or other ischemic process to explain the severe worsening lactic acidosis. The sepsis workup, including blood cultures, was negative. A repeat ABG was taken on day six after surgery, revealing worsening metabolic acidosis with a pH of 7.13, a bicarbonate of 5.9 mmol/L, and a base excess of -21.4 mmol/L, suggestive of type B lactic acidemia. That same day, the patient expired.

## Discussion

Neuroendocrine neoplasms have a current estimated prevalence of approximately 100,000 cases in the United States [5] and comprise fewer than 1% of all newly diagnosed malignancies [6]. These tumors are most commonly found in the gastrointestinal tract (62-67%), followed by the lung (22-27%) [6]. While low-grade neuroendocrine tumors (NETs) are associated with a favorable prognosis, high-grade NECs such as the one our patient had are characterized by their rapid rate of progression [7]. The patient’s diagnosis of SCLC NEC is often associated with lung origin, though other organ systems such as the gastrointestinal system are also possible [8].

Although NETs themselves are not significantly associated with smoking [8], SCLC occurs almost exclusively in heavy smokers and most commonly presents as a large hilar mass with mediastinal adenopathy and significant metastatic spread [9]. The clinical presentation usually includes cough, dyspnea, hemoptysis, weight loss, and debility [10]. Small-cell carcinoma has also been associated with paraneoplastic syndromes such as syndrome of inappropriate antidiuretic hormone secretion, Cushing syndrome, and Lambert-Eaton syndrome [10]. Patients presenting with these symptoms and a smoking history usually undergo CT scans of the chest, and if a suspicious mass is found, a biopsy with histopathology is needed to confirm the diagnosis. Without treatment, the median survival is two to four months [11].

Our patient’s age, sex, and 30-pack/year smoking history placed him at higher risk for NEC (SCLC type), but the absence of pulmonary symptoms in his case is highly unusual. He was also compliant with his yearly chest CT screening, which never showed any suspicious features, and his routine screening lab work, including liver function tests, was always unremarkable. The postoperative chest CT findings of airspace infiltrates and ground-glass opacities in the upper lobes and right middle lobe were inconsistent with the radiologic presentation of SCLC, as 90-95% of SCLCs are found centrally [12].

One plausible hypothesis for the pathophysiology of this patient’s presentation relates to a very quick physiologically and biochemically overwhelming increase of the tumor burden to the liver, involving the gallbladder. As the patient did not have evidence of gallstones or choledocholithiasis, it is possible that the direct hyperbilirubinemia was associated with the involvement and compression of the intrahepatic biliary tree by the quickly and diffusely enlarging tumor bulk. Likewise, transaminitis can be explained by tumor invasion. There were also metastatic emboli found in the liver, gallbladder vasculature, and gallbladder wall on pathology analysis. To date, we are unaware of any other studies or case reports detailing a patient with NEC (SCLC type) presenting in a similar fashion. There are, however, some reports of non-SCLC presenting this way [13-16]. The patient in the most recent of these reports presented similarly with progressively worsening right upper quadrant abdominal pain with positive Murphy’s sign on examination [16]. In that particular case, however, the patient’s liver function tests were within normal limits, and the patient’s liver was grossly normal on CT imaging [16]. The patient had a subclinical metastatic lesion on the brain [16], which was not present in our patient’s case.

The other atypical feature of this case is this patient’s acid-base status. His first ABG revealed a compensated primary metabolic acidosis that was likely chronic with a base excess and an anion gap explained by a severely elevated lactic acidemia. This likely represents lactic acidosis without the presence of systemic ischemia, also known as Type B lactic acidosis. Type B lactic acidosis is a rare paraneoplastic process primarily associated with hematologic malignancies [17]. However, case reports do exist of Type B lactic acidosis associated with solid tumors. One case report conducted a literature review and found that 90% of these solid tumors had some level of liver involvement, postulating that this may be due to either the liver’s role in the metabolism of lactate or its implication in several metastatic pathways [18].

The biochemical causes of severe lactic acidemia in this case are likely twofold. The Ki-67 of nearly 100% indicates a rapidly growing cancer with resulting tumor ischemia and necrosis as well as ischemia of the healthy surrounding native tissue. Additionally, tumor cells have been observed to increase their utilization of glycolysis and subsequent conversion of pyruvate to lactic acid when compared to healthy cells despite adequate oxygen levels in a phenomenon known as the Warburg effect [19]. One study reviewed 14 cases of lactic acidosis due to solid organ malignancies and developed a similar theory for the overproduction of lactic acid in these patients [20]. However, the literature on this topic remains sparse.

## Conclusions

The rapid clinical deterioration of this patient highlights the importance of reporting other similar cases to develop a better understanding of the biology and natural history of these carcinomas, particularly when they present in such an atypical fashion.
